# Therapeutic apheresis: is it safe in children with kidney disease?

**DOI:** 10.1007/s00467-024-06346-0

**Published:** 2024-03-19

**Authors:** Muhammed Doğukan Kalenderoğlu, Elif Çomak, Gülşah Kaya Aksoy, Uğur Bilge, Osman Alphan Küpesiz, Mustafa Koyun, Sema Akman

**Affiliations:** 1 Department of Pediatrics, Faculty of Medicine, Akdeniz University, Antalya, Turkey; 2 Department of Pediatric Nephrology, Faculty of Medicine, Akdeniz University, Antalya, Turkey; 3 Department of Biostatistics and Medical Informatics, Faculty of Medicine, Akdeniz University, Antalya, Turkey; 4 Pediatric Hematology and Oncology, Faculty of Medicine, Akdeniz University, Antalya, Turkey

**Keywords:** Plasma exchange, Immunoadsorption, Complication, Kidney disease

## Abstract

**Background:**

Therapeutic apheresis (TA) is already used to treat various diseases in the field of nephrology. The aim of this study was to evaluate the frequency and types of complications that occur during TA in children with kidney disease.

**Methods:**

Records of children (≤ 18 years) who underwent TA between 2007 and 2022 were retrospectively reviewed. Children with missing data and those with a diagnosis of nonnephrological disease were excluded.

**Results:**

A total of 1214 TA sessions, including 1147 therapeutic plasma exchange (TPE) sessions and 67 immunoadsorption (IA) sessions, were performed on the 108 patients enrolled in the study. Forty-seven percent of the patients were male, and the mean age was 12.22 ± 4.47 years. Posttransplant antibody-mediated rejection (64.8%) and hemolytic uremic syndrome (14.8%) were the most common diagnoses indicating TA. Overall, 17 different complications occurred in 58 sessions (4.8%), and 53 sessions (4.6%) were not completed because of these complications. The distribution of complications among the patients was as follows: 41.4% had technical complications, 25.9% had allergic complications, and 32.7% had others. The most common technical complication was insufficient flow (37.5%). The incidence of complications was greater in patients aged 3–6 years than in patients in the other age groups (*p* = 0.031). The primary disease, type of vascular access, and rate of fresh frozen plasma/albumin use were similar between patients with and without complications (*p* values of 0.359 and 0.125 and 0.118, respectively).

**Conclusions:**

Our study showed that complications occurred in only 4.8% of TA sessions. The most common complication was technical problems.

**Graphical Abstract:**

**Figure:**
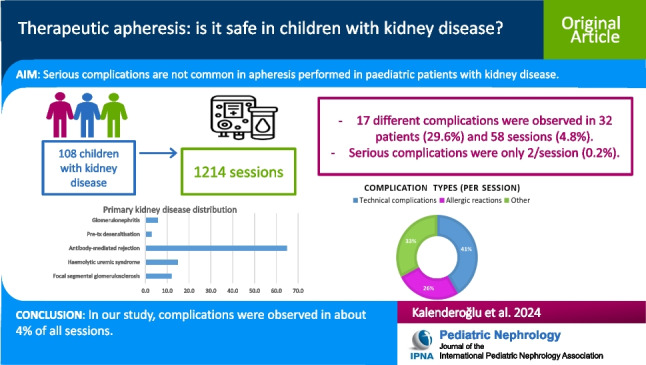
A higher resolution version of the Graphical abstract is available as Supplementary information

**Supplementary Information:**

The online version contains supplementary material available at 10.1007/s00467-024-06346-0.

## Introduction

Therapeutic apheresis (TA) is a treatment used to remove pathogens from a patient’s blood. Two different technological methods are available: centrifugation and membrane filtration [1, 2]. Apheresis procedures may be preferred in the treatment of various medical conditions, such as rheumatologic diseases, neurologic and hematologic disorders, kidney diseases, and poisoning, as well as in the management of patients with multiple-organ failure.

Kidney diseases for which TA is commonly used include steroid-resistant nephrotic syndrome, thrombotic microangiopathy (TMA), systemic lupus erythematosus (SLE), antineutrophil cytoplasmic antibody (ANCA)-associated vasculitis (AAV), anti-glomerular basement membrane (anti-GBM) disease, C3 glomerulopathy, and humoral rejection in kidney transplant patients [2].

According to a retrospective review of 58 pediatric patients with kidney disease from Ireland, the primary disease was hemolytic uremic syndrome in 72.4% of patients [3]. In a study by Joseph et al. analyzing 641 therapeutic plasma exchange (TPE) procedures in 47 pediatric patients, antibody-mediated rejection and bone marrow transplant-associated thrombotic microangiopathy were the most frequently indicated diseases [4]. According to a study of data from 14 centers in 12 European Union countries, the most common diagnoses were focal segmental glomerulosclerosis (FSGS), hemolytic uremic syndrome (HUS), and pretransplant desensitization [5]. In this study of 80 pediatric patients, TPE was performed in 67 children, immunoadsorption (IA) in 10 children, and double filtration plasmapheresis (DFPP) in 3 children [5]. The IA process uses special bioactive membranes with binding capacity for the substance to be removed. For this reason, IA is described as a more selective and antigen-specific protocol; however, it is only used for selected indications because of its cost [6, 7].

A retrospective study of 4004 TA procedures performed on 298 pediatric patients; mostly those with nephrological and neurological diseases revealed that complications occurred in 11% of all sessions [8]. This study revealed that IA was associated with fewer complications than the other methods [8]. In a study analyzing only plasma exchange and including 1201 procedures in pediatric patients, the complication rate was 12.7% [9]. In the study by Taylan et al., it was observed that younger age was more strongly associated with catheter dysfunction, but TA-related complications were more frequent, especially in the adolescent age group [8]. Vascular access is the most important step in accessing the TA in pediatric patients, and age may be a determining factor in the choice of treatment, especially when hemodynamic instability is considered.

However, few studies have evaluated the safety of TA in pediatric patients with kidney disease. The aim of this study was to investigate the distribution of nephrological diagnoses indicating TA treatment in pediatric patients, the frequency of complications, and the impact of complications on treatment sustainability.

## Materials and methods

Between 1 January 2007, and 31 August 2022, the records of patients aged ≤ 18 years who underwent TA at the Akdeniz University Faculty of Medicine were retrospectively reviewed. In our clinic, the American Society for Apheresis (ASFA) criteria are the basis for indications for TA [10]. Among these indications, patients with a diagnosis of kidney disease were included in the study. Based on the ASFA 2019 criteria, the IA procedure is used in cases of antibody-mediated rejection and uncontrolled immune thrombocytopenia in SLE patients [10]. The study exclusion criteria were patients with non-kidney disease or patients with missing medical data. This study was approved by the Akdeniz University Faculty of Medicine Hospital Clinical Research Ethics Committee.

Patient demographic and clinical characteristics were obtained from the electronic hospital database; patient medical records, clinical data, and complications were collected by apheresis unit nurses. Anthropometric measurements, primary kidney disease diagnosis, treatments administered before TA, number of sessions, vascular access, replacement and anticoagulant fluids used, preprocedure laboratory values, and complications observed during the procedure were documented for each patient.

### Characteristics of therapeutic apheresis procedures

The estimated plasma volume was calculated using the formula 80 × kg × (1 − Ht)/100. At our center, we replace 1–1.5 times the estimated plasma volume as a routine practice. This approach can achieve a replacement rate of 15–20 mL/kg/h at a blood flow rate of approximately 3–5 mL/kg/min. Fresh frozen plasma (FFP) or 4% albumin was preferred as the replacement fluid. Peripheral veins, central venous catheters, or arteriovenous fistulae (AVFs) were preferred for vascular access. Priming was performed in a selected group of patients: those with a body weight less than 30 kg, an extracorporeal volume greater than 10–15% of the total blood volume, or a hematocrit level predicted to fall below 25% during the procedure. Priming was performed with red blood cells.

As an anticoagulant treatment, acid-citrate-dextrose A (ACDA) was used in TPE, and heparin was used for immunoadsorption. Prior to all procedures, hemoglobin and serum calcium levels were assessed. All the calcium data analyzed in the study were corrected for the serum albumin concentration. If the Hb level was < 8 g/dL, an erythrocyte transfusion was administered, and intravenous calcium gluconate replacement (0.2 mmol/kg/dose) was given to patients with Ca levels < 8 mg/dL. For premedication, pheniramine (1 mg/kg) and methylprednisolone (1 mg/kg) were administered routinely.

Complications were categorized into three groups: technical complications, allergic reactions, and other complications. Technical complications include insufficient flow, device malfunction, blood leakage, vascular access problems, and thrombus. Allergic reactions were categorized as urticaria, pruritus, angioedema, or anaphylaxis. Nonspecific clinical symptoms such as abdominal pain, hypertension, cramps, nausea/vomiting, hypotension, chest pain/palpitation, headache, and fever were classified in the other group.

### Statistical analysis

The data were analyzed using IBM SPSS Statistics 23 © Copyright SPSS, Inc. The normality of continuous variables was assessed using the Kolmogorov‒Smirnov test. Categorical variables are expressed herein as the frequency (*n*) and percentage (%), while continuous variable data conforming to parametric assumptions are presented as the mean ± standard deviation (SD), and those not conforming to parametric assumptions are expressed as median (IQR) values. Categorical variables were evaluated using Pearson’s chi-square test and Fisher’s exact test, with Bonferroni corrections applied. For independent variables for which parametric assumptions were not met, comparisons were evaluated using the Mann‒Whitney *U* test. A statistical significance level of 0.05 was used.

## Results

### Patient and session information

**Fig. 1:**
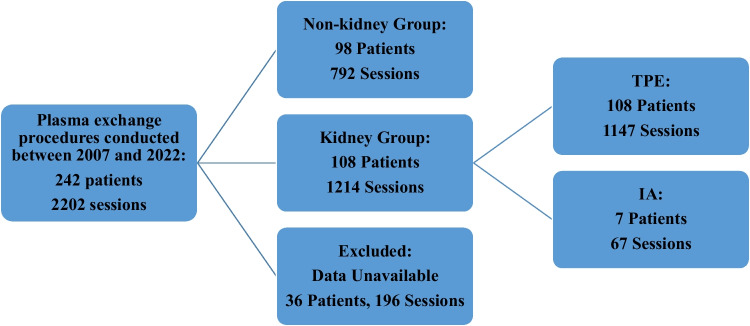
Flow chart of the study. *TPE*, therapeutic plasma exchange; *IA*, immunoadsorption

A total of 2202 apheresis sessions were performed on 242 pediatric patients. Of these, 196 sessions in 36 patients were excluded because of insufficient medical records. In 792 sessions performed in 98 patients, the indicated diagnosis was nonnephrological disease. Therefore, a total of 108 patients (52.4%) with a diagnosis of kidney disease and 1214 sessions (60.5%) were included in the study (Fig. 1).

### Characteristics of pediatric kidney patients who underwent apheresis

The mean age of the patients was 12.22 ± 4.47 years, and 47% (*n* = 51) of them were male. Half of the study group (50%) consisted of children aged 6–15 years. Additionally, 11 patients (10.2%) were less than 3 years old. The median weight was 38.12 kg (IQR 23.00–56.00); the median height was 143.50 cm (IQR 124.50–161.00), and the median BMI was 18.65 kg/m^2^ (IQR 16.44–21.50) (Table 1). Among the apheresis patients, 58.3% were between 30 and 70 kg, 31.5% were between 10 and 30 kg, and only 4 children (3.7%) were under 10 kg.

**Table 1:** Patient demographic characteristics and apheresis data (*n* = 108)

Variable	Values
Male gender (%)	51 (47)
Mean age (year)	12.22 ± 4.47
Median weight (kg) (IQR)	38.12 (23.00–56.00)
Median weight (cm) (IQR)	143.50 (124.50–161.00)
Median BMI (kg/m^2^) (IQR)	18.65 (16.44–22.50)
Median number of sessions (IQR)	5 (3.00–14.00)
Primer kidney disease diagnosis (%)	
Focal segmental glomerulosclerosis	13 (12.0)
FSGS Post Tx FSGS Hemolytic uremic syndrome Typical HUS Atypical HUS Pretransplant desensitization	3 (2.8) 10 (9.3) 16 (14.8) 6 (5.6) 10 (9.3) 3 (2.8)
Acute antibody-mediated rejection	70 (64.8)
Glomerulonephritis	6 (5.6)
C3 glomerulopathy	3 (2.8)
Rapidly progressive glomerulonephritis	2 (1.9)
Lupus nephritis	1 (0.9)
ASFA (%)	
Category 1 Category 2 Category 3 Category 4 Undefined	92 (85.2) - 12 (11.1) - 4 (3.7)
Hypocalcemia (%)	
< 8 mg/dL ≥ 8 mg/dL No data	120 (9.9) 971 (80.0) 123 (10.1)
Median Hb (gr/dL) (IQR)	9.60 (5.70–18.70)
Low hemoglobin levels (%)	
< 8 g/dL ≥ 8 g/dL No data	203 (16.7) 1002 (82.5) 9 (0.7)
Treatment modality according to the number of patients (%)	
TPE IA	108 (100) 7 (6.4)
Treatment method according to the number of sessions (%)	
TPE IA	1147 (94.5) 67 (5.5)

*BMI* body mass index, *FSGS* focal segmental glomerulosclerosis, *HUS* hemolytic uremic syndrome, *IA* immunoabsorption, *TPE* therapeutic plasma exchange, *Tx* transplant, *ASFA* American Society for Apheresis

The TA procedure was administered as follows: 94.5% (*n* = 1147) of all procedures involved TPE, and 5.5% (*n* = 67) involved IA. The detailed technical specifications of the TPE and IA procedures are presented in Table 2. The median flow rate and volume processed were greater in patients with IA than in those receiving TPE (*p* < 0.001 and *p* < 0.001, respectively). Among all the apheresis procedures, 1167 (96.1%) procedures were performed at the apheresis unit, whereas 47 (3.9%) procedures were performed at the pediatric intensive care unit.

**Table 2:** Technical features of plasma exchange and immunoabsorption

	Plasma exchange	Immunoabsorption
Median duration time (minute) (IQR)	80.0 (65.0–99.0)	50.0 (37.0–60.0)
Median flow rate (mL/min) (IQR)	50.0 (40.0–60.0)	120.0 (100.0–150.0)
Median total blood volume (mL) (IQR)	3466.0 (2500.0–4520.0)	6000.0 (3600.0–9500.0)
Median exchanged plasma volume (mL) (IQR)	1839.0 (1356.5–2487.5)	NA
Replacement solution (%)		
FFP %4 albumin	1066 (87.8) 81 (6.7)	NA NA
Anticoagulation (%)		
Heparin ACDA	NA 1147 (94.5)	67 (5.5) NA
Vascular access (%)		
Peripheric access Central venous catheters Arteriovenous fistulas	131 (10.8) 977 (86) 39 (3.2)	- 67 (100) -

*FFP* fresh frozen plasma, *ACDA* anticoagulant citrate dextrose solution A

The distribution of diagnoses indicating TA was as follows: posttransplant antibody-mediated rejection in 70 (64.8%) patients, hemolytic uremic syndrome (HUS) in 16 (14.8%) patients, focal segmental glomerulosclerosis in 13 (12.0%) patients, glomerulonephritis in 6 (5.6%) patients, and pretransplant desensitization in 3 (2.8%) patients (Table 1). Of the 7 patients who underwent IA, 6 had antibody-mediated rejection after transplantation, and 1 had SLE-associated immune thrombocytopenia. The median number of sessions by primary diagnosis was as follows: FSGS 7.50 (IQR 4.25–2.25), typical HUS 5.00 (2.75–8.00), atypical HUS 7.50 (3.50–12.75), desensitization 9.00 (5.00–12.00), antibody-mediated rejection 7.00 (IQR 5.00–13.00), and glomerulonephritis 6.00 (IQR 2.50–11.00).

Frozen plasma was used as the replacement solution in 87.8% of patients, while albumin was used in 6.7% of patients. The most commonly used anticoagulant was citrate (94.5%), and 5.5% of patients received heparin. Central venous catheters were used for vascular access in 79.8% of patients and arteriovenous fistulas in 4.6%, and the remaining 15.6% of patients had suitable peripheral veins for apheresis procedures (Table 2).

According to our laboratory evaluation, hypocalcemia was detected in 120 sessions (9.9%) prior to the TA procedure. Additionally, hemoglobin levels less than 8 g/dL were observed in 203 sessions (16.7%), and erythrocyte transfusions were administered to all of these patients (Table 1).

### Frequency and distribution of complications

A total of 17 different complications were observed in 58 (4.8%) of the 32 patients (29.6%). None of the IA procedures had complications. A total of 53 sessions (4.4%) could not be completed due to complications. Of the 1214 sessions, technical problems were recorded in 2.0% (*n* = 24) of sessions, allergic reactions in 1.2% (*n* = 15), and other complications in 1.6% (*n* = 19). The most common technical complication was insufficient flow (15.5%, *n* = 9 sessions), and the most common allergic complication was urticarial rash (19%, *n* = 11 sessions) (Table 3). Only 2 sessions (0.2%) had life-threatening serious complications, and both were anaphylactic reactions. These two patients were diagnosed with posttransplant antibody-mediated rejection, and FFP was used as the replacement fluid. Adrenaline was administered to both patients. Toward the end of the session, both patients developed pruritic urticarial rash, angioedema, and wheezing; with the onset of respiratory distress, the procedures of these patients judged to be in anaphylaxis were stopped, oxygen support was provided, and IM epinephrine was administered. One patient’s procedures were continued with IA, and the other patient’s treatment was changed to pharmacological treatment methods. The apheresis procedure was stopped, and the clinical condition of these patients was controlled without the need for intensive care, with no further continuation of apheresis treatment.

**Table 3:** Distribution of complications according to the number of apheresis sessions (*n* = 58)

	Complication	*n*	% (rate of all complications)	% (subgroup ratio)
Technical complications (*n* = 24, 41.4%)	Insufficient flow Device malfunction Blood leakage Vascular access problem Thrombus	9 5 5 4 1	15.5 8.6 8.6 6.9 1.7	37.5 20.8 20.8 16.7 4.2
Allergic complications (*n* = 15, 25.9%)	Urticaria Pruritus Angioedema Anaphylaxis	11 2 2 2	19 3.4 3.4 3.4	73.3 8.3 8.3 8.3
Others (*n* = 19, 32.7%)	Abdominal pain Hypertension Cramps Nausea/vomiting Hypotension Chest pain/palpitations Headache Fever	4 3 3 2 2 1 1 1	6.9 5.2 5.2 3.4 3.4 1.7 1.7 1.7	21.1 15.8 15.8 10.5 10.5 5.2 5.2 5.2

Complications were more common in children aged 3–6 years and least common in children aged 6–15 years (*p* = 0.031). In contrast, the difference was similar between the weight groups (*p* = 0.655). Moreover, there was no difference in the frequency of complications between primary kidney disease diagnoses and native kidney patients/transplant recipients (*p* = 0.359 and *p* = 0.526, respectively). The incidence of complications was similar between vascular access types and fluid replacement options (*p* = 0.270 and *p* = 0.118, respectively). The pre-apheresis hemoglobin level was greater in the complication session than in the other sessions, but the ratio of patients with a hemoglobin level < 8 g/dL was similar (*p* = 0.004 and *p* = 0.530). In addition, the hemoglobin levels measured in the patients’ first session were similar (10.07 ± 2.04 vs. 9.49 ± 1.79, *p* = 0.146).

## Discussion

In our unit, 52.4% of patients in the pediatric age group who underwent apheresis had a diagnosis of kidney disease. In the study of 80 pediatric patients from 14 different centers, 56 patients were followed up in the nephrology department [5]. Similar to the findings of our study, HUS, antibody-mediated rejection, and FSGS are among the most frequently reported diseases in the literature [4].

In our study, complications were observed in only 4.8% of all sessions in children receiving TA for kidney disease. The rate of serious life-threatening complications was 0.2%, and both cases were severe anaphylaxis. The incidence of complications associated with TA varies between 4 and 55% in different series [5, 8, 9, 11–16]. Lu et al. reported a complication rate of 12.7% in their study of 435 pediatric patients who underwent 1202 apheresis sessions [9]. In a study including pediatric patients, most of whom had kidney disease; the frequency of minor complications was found to be 6.9% in the TPE group and 9.7% in the IA/DFPP group [5]. However, a study by Taylan et al. showed a greater complication rate with TPE procedures than with IA procedures (12.2% vs. 9.5%, *p* < 0.001) [8]. The complication rate observed in our study was lower than that reported in the literature, and no complications occurred in the IA group. However, we believe that this may be attributed to the relatively low number of patients undergoing immunoadsorption and the preference for IA in selected cases due to cost concerns in recent years.

The most common complication associated with apheresis (41.4%) was technical problems. However, the incidence of allergic reactions was 25.9%. In an adult study by Shemin et al., complications were reported in 614 of 1727 sessions (36%), and the most common complications were fever (7.7%), urticaria (7.4%), and hypocalcemia (7.3%) [17]. In an Austrian pediatric study, complications were reported in 31 out of 280 sessions (11.0%), and the most common problem was vascular access failure [14]. In two studies evaluating the use of apheresis at pediatric intensive care units, the most common complications were circuit blockage (7.2% and 6.4%) and vascular access problems (4.8% and 4.9%) [16, 18]. In our study group, 3.9% of the sessions took place in the intensive care unit.

In our study, albumin was used for replacement in 6.7% of therapeutic plasma exchange sessions, and no allergic reactions were observed in patients receiving albumin. In the study by Lu et al., the most common complication was pruritus and urticaria, occurring in 7% of patients [9]. Joseph et al. evaluated 335 apheresis sessions, and no allergic reactions were observed in sessions using albumin, whereas 13.4% of allergic reactions were observed in sessions using FFP [4]. Runowski et al. used FFP as a replacement fluid in 74.7% of 389 apheresis sessions in 59 pediatric kidney transplant patients and reported an allergic reaction rate of 1%. All patients who developed allergic reactions were found to receive FFP [19].

The most common age group treated with apheresis for kidney disease was 6 to 15 years, and 5.9% of all apheresis sessions were in children under 3 years of age. In a study by Sık et al. analyzing patients treated in the pediatric intensive care unit between 2015 and 2019, the median age of the 135 patients who underwent TPE was 34 months (10–108), and the incidence of minor complications was 16.3% [16]. Taylan et al. reported an increased complication rate in children > 14 years of age compared to those aged 9–13 years in a study of 298 children and adolescents [8]. However, in our study, the most common complication was observed between the ages of 3 and 6 years.

In our study, hemoglobin levels were greater in sessions with complications, but the frequency of complications was similar between the hemoglobin < 8 g/dL and > 8 g/dL groups. As the initial hemoglobin levels of the patients were similar, the high hemoglobin levels observed in the sessions with complications suggested that these patients may have had repeated procedures. Although a clear relationship between hemoglobin concentration and the incidence of complications has not been reported in the literature, Michon et al. identified a hemoglobin level of 7 g/dL as posing a risk for the development of complications [11].

Our study has several limitations. First, it reflects a single-center experience and is retrospective in nature. The limited number of patients in the IA group did not allow for a balanced comparison. The medical treatments used for primary kidney diseases were not recorded, which is another limitation. However, this study contributes to the literature by evaluating pediatric patients who underwent apheresis and were diagnosed with kidney disease. In the future, multicenter and prospective studies will shed light on the sustainability and safety of apheresis treatment in nephrology and provide valuable insights for clinicians.

## Conclusions

The incidence of serious complications associated with apheresis in pediatric patients with kidney disease is quite low. The most common complications encountered were technical problems, followed by allergic reactions. Among pediatric patients, those aged 6 to 15 years appear to have the highest incidence of complications, while those under 3 years of age do not have a significantly different rate of complications.

### Supplementary Information

Below is the link to the electronic supplementary material.

Graphical Abstract (PPTX 255 KB)

## Data Availability

The data that support the findings of this study are available from the corresponding author [M.D.K.].
