# Supplementary material for: Therapeutic apheresis: is it safe in children with kidney disease?
Source: Pediatr Nephrol. 2024 Mar 19;39(8):2451–7. doi: 10.1007/s00467-024-06346-0 (PMC11199252; doi:10.1007/s00467-024-06346-0)
Supplement: Supplementary file 1 — Graphical Abstract (PPTX 255 KB) [file 467_2024_6346_MOESM1_ESM.pptx]

## Slide 1
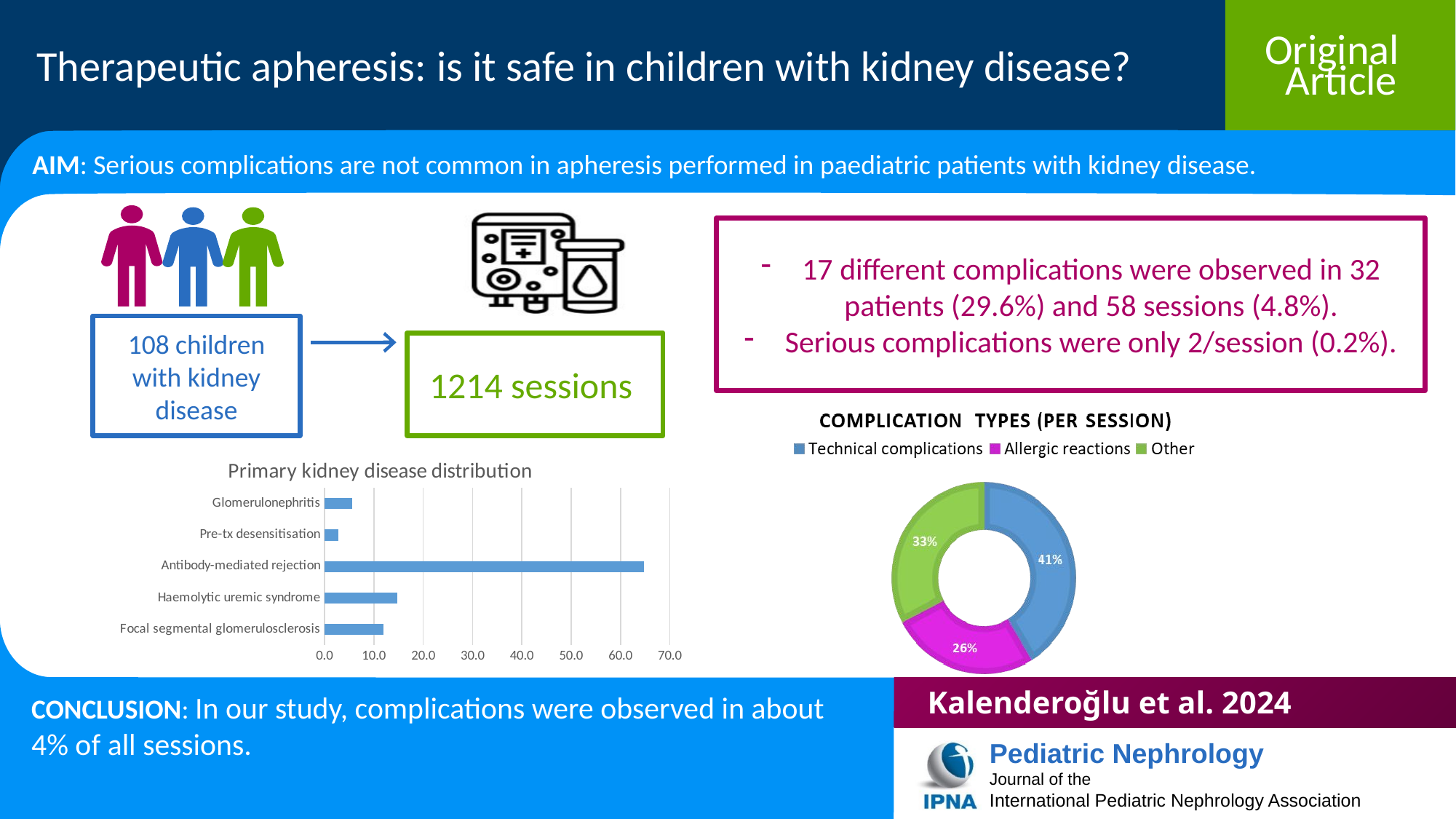

Therapeutic apheresis: is it safe in children with kidney disease?
AIM: Serious complications are not common in apheresis performed in paediatric patients with kidney disease.
17 different complications were observed in 32 patients (29.6%) and 58 sessions (4.8%).
Serious complications were only 2/session (0.2%).
108 children with kidney disease
1214 sessions
### Chart: Primary kidney disease distribution
| Category | |
|---|---|
| Focal segmental glomerulosclerosis | 12.0 |
| Haemolytic uremic syndrome | 14.8 |
| Antibody-mediated rejection | 64.8 |
| Pre-tx desensitisation | 2.8 |
| Glomerulonephritis | 5.6 |Kalenderoğlu et al. 2024
CONCLUSION: In our study, complications were observed in about 4% of all sessions.
